# In-Situ Sulfuration of CoAl Metal–Organic Framework for Enhanced Supercapacitor Properties

**DOI:** 10.3390/ma17164030

**Published:** 2024-08-13

**Authors:** Mengchen Liao, Kai Zhang, Chaowei Luo, Guozhong Wu, Hongyan Zeng

**Affiliations:** 1 School of Chemistry and Chemical Engineering, Central South University, Changsha 410083, China; 2 Shanghai Institute of Applied Physics, Chinese Academy of Sciences, Shanghai 201800, China; 3 College of Chemical Engineering, Xiangtan University, Xiangtan 411105, China

**Keywords:** amorphous/crystalline heterophase, Al metal–organic framework, cobalt sulfide, supercapacitor

## Abstract

Designing efficient electrode materials is necessary for supercapacitors but remains highly challenging. Herein, cobalt sulfide with crystalline/amorphous heterophase (denoted as Co(Al)S) derived from an Al metal–organic framework was constructed by ion exchange/acid etching and subsequent sulfidation strategy. It was found that rational sulfidation by adjusting the sulfur source concentration to a suitable level was favorable to form a 3D nanosheet-interconnected network architecture with a large specific surface area, which promoted ion/electron transport and charge separation. Benefiting from the features of the unique network structure and heterophase accompanied by aluminum, nitrogen and carbon coordinated in amorphous phase, the optimal Co(Al)S_(10)_ exhibited a high specific capacity (1791.8 C g^−1^ at 1 A g^−1^), an outstanding rate capability and an excellent cycling stability. Furthermore, the as-assembled Co(Al)S//AC device afforded an energy density of 72.3 Wh kg^−1^ at a power density of 750 W kg^−1^, verifying that the Co(Al)S was a promising material for energy storage devices. The developed scheme is expected to promote the application of MOF-derived electrode materials in electrochemical energy storage and conversion fields.

## 1. Introduction

Supercapacitors have been exploited as promising energy storage devices owing to their high power density, fast charge/discharge rates, low cost and high safety, but their relatively lower energy density than other storage devices (i.e., batteries and conventional capacitors) still restricts their large-scale application [1,2]. Consequently, ongoing research on electrochemical supercapacitors is mainly focused on customizing electrode materials with high capacitance, to promote the sustainable development of electrochemical energy storage systems [3,4]. Up till now, transition metal compounds, such as transition metal oxides/hydroxides [5,6], transition metal selenides [7] and transition metal sulfides [8,9], have been studied as promising alternatives for electrode materials with high energy densities. In this regard, transition metal sulfides (TMSs) have been extensively explored as electrode materials due to their high theoretical capacity, low cost and facile synthesis. However, the low rate capability and the limited cyclic life of TMSs critically preclude their utilization in practical supercapacitors [10,11,12]. Therefore, it is essential to get desirable electrochemical performance such as greater conductivity and higher capacitance and rate capability.

In recent years, metal–organic frameworks (MOFs) have been proven to be the ideal precursors or sacrificial templates to prepare metal sulfides with enhanced reversible capacity and good rate capability and cycle performance in supercapacitors. The components (N, C, S, etc.) in MOFs can be evenly doped into the MOF-derived materials, which further improves the electrochemical properties [4,12,13,14,15,16]. For example, ZIF-67-derived NiCoMn prepared via etching/ion-exchange reaction and sulfurization exhibited a high specific capacitance of 2098.2 F g^−1^ at 1 A g^−1^, and the corresponding as-assembled NiCoMn-S//activated carbon (AC) device achieved an energy density of 50.0 Wh kg^−1^ at 850.0 W kg^−1^ and a capacitance retention of 73.6% after 6000 cycles [17]. Ni-MOF-derived NiS@C with S-vacancies through ion exchange and calcination showed a high reversible specific capacity (1728 F g^−1^, 1 A g^−1^), stable cycling (72% capacity retention over 8000 cycles) and a satisfying rate capability. The corresponding NiS@C//AC device provided an energy density of 36.88 Wh kg^−1^ at 750 W kg^−1^ [18]. NiCo-MOF-derived sulfides on NF (HN_2_CMS) presented a specific capacitance of 938.1 C g^−1^ at 1 A g^−1^ and good cycling stability (85.0% after 5000 cycles), while the HN_2_CMS//AC device provided an energy density of 51.2 Wh kg^−1^ at 800 W kg^−1^ [19]. Despite these progresses, supercapacitor performances remain to be further improved.

On another front, phase engineering has been proven as an efficacious tactic for modulating the physicochemical properties and augmenting the electrochemical performance of electrode materials [20,21]. Among these, the profound synergy between amorphous and crystalline (a/c) components, which exerted respective superiorities and avoided the shortcomings of amorphous- and crystalline-phase structures, and thus showed unusual features in electrochemical processes, has captured the attention of researchers [22,23]. As a special type of phase structure, the amorphous phase has an isotropic nature, inherent abundant defects and no grain boundaries due to the long-range disordered but short-range ordered arrangement of the atoms, endowing multiple percolation pathways and isotropic ion diffusion channels to offer fast ion diffusion and high active site density and charge storage. However, the intrinsically lower conductivity than that of the crystalline counterpart depresses the application of amorphous material [24,25]. On the contrary, the crystalline phase with defect-less, long-range order and anisotropy can offset the above shortages due to better mechanical stability and faster electron/ion transport than the amorphous phase [21,26]. Therefore, constructing the a/c heterophase structure is a feasible strategy to attain excellent cycling stability and rate capability for electrode materials in supercapacitor applications. 

Concerning the above-mentioned investigations and our previous works, cobalt sulfide (Co(Al)S) with aluminum, nitrogen and carbon coordinated amorphous/crystalline heterophase structure was designed and prepared through a self-templated CAU-1 approach. The Co(Al)S was synthesized by ion exchange/acid etching the CAU-1 template on NF into the CoAl_2_O_4_ intermediate followed by sulfuration, where the CAU-1 template was also a nitrogen and carbon source. Through various characterization and electrochemical measurements, it was found that rational sulfidation by adjusting the sulfur source concentration to a suitable level was favorable to form a 3D nanosheet-interconnected network architecture with a large specific surface area, which gave rapid ion/electron transport, high electrical conductivity, satisfactory rate capability and excellent specific capacity. Furthermore, a laboratory-scale hybrid supercapacitor (HSC) was fabricated using the Co(Al)S_(10)_ and activated carbon as positive and negative electrodes, which delivered superior energy and power densities.

## 2. Experimental

### 2.1. Chemicals and Materials

NH_4_F (99%, Sigma-Aldrich, Shanghai, China), activated carbon (AC, YP50F, Kuraray, Shanghai, China), 2-amino-terephthalic acid (NH_2_-BDC, 99%, Sigma-Aldrich) and other chemical reagents (AR, Nan-Jin Chemical Reagent Co., Ltd., Nanjin, China) were employed without further purification, and deionized water was used to prepare the solutions. Nickel foam (NF, 97.2% porosity, 99.8% purity) was purchased from Hunan Corun New Energy Co., Ltd. (Tianjin, China). Prior to utilization, the NF (4 cm × 2 cm × 1 mm) was cleaned in 3 mol L^–1^ HCl solution under ultrasonic conditions for 15 min and then rinsed for use. 

### 2.2. Materials Synthesis

Synthesis of Al-MOF on NF: The Al-MOF (CAU-1) was prepared by a solvothermal method. AlCl_3_·6H_2_O (2.190 g) and NH_2_-BDC (0.548 g) were dissolved in 30 mL methanol. The mixed solution and a precleaned NF (1 × 1 cm^2^) were transferred into a Teflon-lined autoclave and kept at 125 °C for 5 h. After cooling, the resulting product was rinsed and dried to obtain the CAU-1. The mass of the CAU-1 on the NF was about 3.0 mg cm^−2^ according to the mass before and after preparation.

Synthesis of CoAl oxide on NF: A total of 40 mL Co(NO_3_)_2_ solution (7.0 mmol L^−1^) containing a piece of the CAU-1 was transferred into an autoclave and kept at 150 °C for 6 h. The product was rinsed and then dried to gain the CoAl oxide intermediate (CoAlO). The mass of CoAlO on NF was still about 3.0 mg cm^−1^.

Synthesis of CoAl sulfide: A total of 40 mL Na_2_S solution containing a piece of the CoAlO was transferred into an autoclave and kept at 120 °C for 10 h. The resulting product was rinsed and dried and denoted as Co(Al)S. For ease of expression, the Co(Al)S samples prepared in 5.0 × 10^−2^, 7.5 × 10^−2^, 10.0 × 10^−2^ and 15.0 × 10^−2^ mol L^–1^ of Na_2_S were sequentially labeled as Co(Al)S_(5.0)_, Co(Al)S_(7.5)_, Co(Al)S_(10)_ and Co(Al)S_(15)_. The mass of Co(Al)S on NF was about 3.0 mg cm^−1^.

### 2.3. Characterizations and Electrochemical Measurements

Full details for the characterization and electrochemical measurements are provided in the Supporting Information. To avoid the effect of NF, the Co(Al)O and Co(Al)S powders were stripped from NF by longtime ultrasonication and collected for TEM/EDS, XRD, Raman, N2 sorptometry and XPS analyses.

## 3. Result and Discussion

### 3.1. Synthesis of Materials

**Scheme 1:**
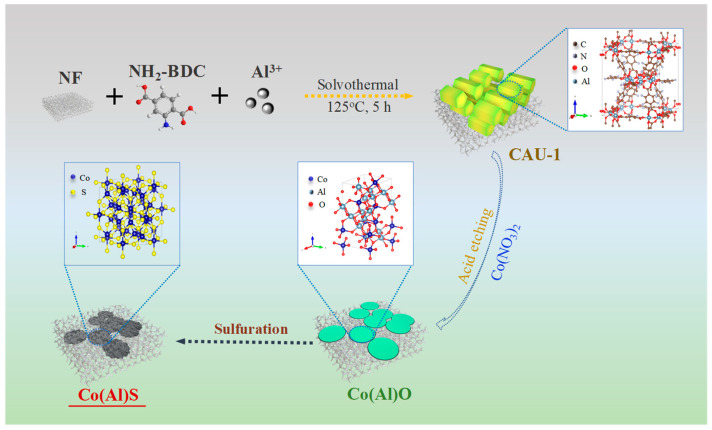
Synthesis procedure and phase transformation of the Co(Al)S.

The preparation of the cobalt sulfides is illustrated in Scheme 1, where methanol, 2-amino-terephthalic acid (NH_2_-BDC) and NF were used separately as solvent, functional ligand and support by mixing metal salts to construct the CAU-1 sacrificial template via a solvothermal method. Subsequently, the Co(Al)O intermediate was prepared through ion exchange/acid etching in Co(NO_3_)_2_ solution, and then the Co(Al)O intermediate was sulfurized into cobalt sulfide with amorphous/crystalline heterophase (Co(Al)S) using Na_2_S as the sulfur source. In this strategy, the CAU-1 template was also a nitrogen and carbon source, while the physicochemical and electrochemical properties were tailored by adjusting the concentration of the sulfur source.

### 3.2. Structural Characterization of Materials

**Figure 1:**
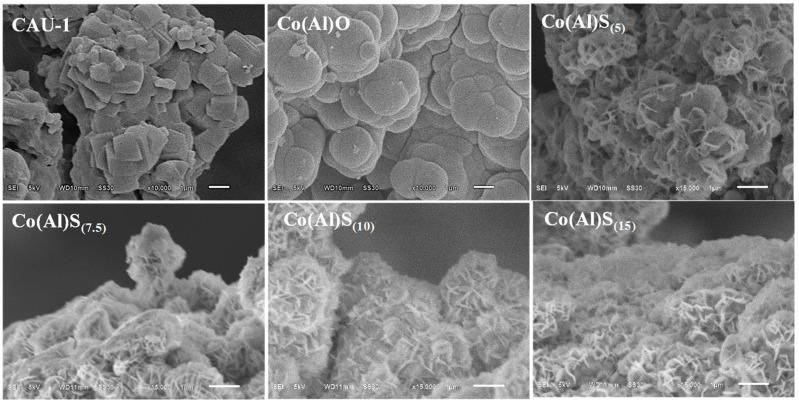
SEM images of the CAU-1, Co(Al)O, and Co(Al)S samples.

The morphology and chemical components of the CAU-1, the Co(Al)O intermediate and the Co(Al)S were observed by SEM/EDS (Figure 1 and Figures S1–S3). The CAU-1 presented a cuboid-shaped structure with a smooth surface, while the Co(Al)O intermediate showed a pie-shape layered structure after ion exchanging/etching the CAU-1 template. When the intermediate was sulfurized into the corresponding cobalt sulfide, the Co(Al)S still kept the 2D layered structure, and the thickness of the nanosheets became thinner with increasing the sulfur source concentration (Figure S1). The Co(Al)S indicated a flower-like “sand-rose” architecture composed of individually 2D nanosheets at low Na_2_S concentration (≤10.0 × 10^−2^ mol L^–1^), but the nanosheets of the Co(Al)S_(15)_ tended to collapse and aggregate at a high concentration (15.0 × 10^−2^ mol L^–1^). Clearly, the 3D flower-like “sand-rose” architecture of the Co(Al)S_(10)_ was composed of individual, looser, and thinner nanosheets, leading to a larger surface area to expose more active sites for accelerating electron transfer (consistent with N_2_ sorptometry results below). As illustrated in Figures S2 and S3, the SEM-EDS pattern and the EDS-mapping images revealed that the Co(Al)S_(10)_ contained N, C, O, Co, Al and S elements, and that the distribution of all the elements was relatively homogeneous in the selected area. The O element arose from the adventitious O species in air, and the existence of C and N elements implied that C and N species were introduced into the Co(Al)S_(10)_ matrix.

**Figure 2:**
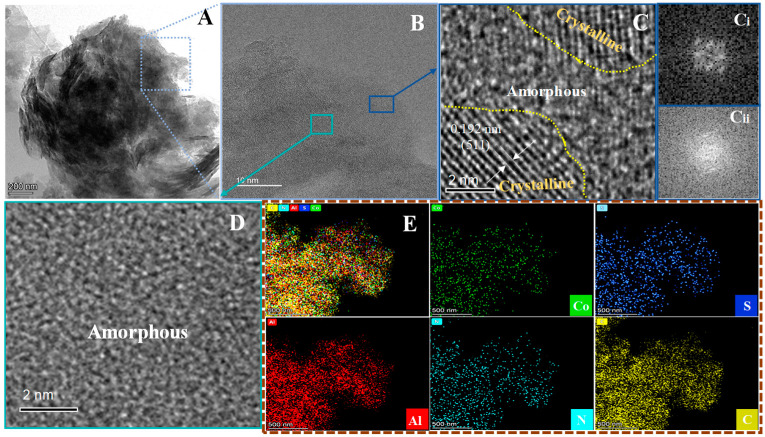
TEM (**A**), HRTEM (**B**–**D**) and HAADF-STEM (**E**) images of the Co(Al)S_(10)_.

The detailed morphology and microstructure of the Co(Al)S_(10)_ were further determined using TEM (Figure 2). The low-magnification TEM image provided direct evidence that ultrathin nanosheets staggered interactively to form a 3D nanosheet-interconnected network architecture. The 3D porous network architecture could expose enough active sites for Faradaic reaction to accelerate electron/ion transfer. The interconnected amorphous and crystalline domains with clear boundaries (yellow border in blue frame) were observed in the HRTEM images (Figure 2B,C). The lattice space of 0.192 nm was related to the (511) crystal plane for cubic Co_9_S_8_ (JCPDS, no. 19-0364; Figure 2C), and the amorphous domains presented fuzzy and random atomic arrangement (Figure 2D), confirming that the Co(Al)S was a crystalline/amorphous heterophase structure. The heterophase feature was further verified by the corresponding fast Fourier transformation (FFT) patterns at the selected areas, in which the bright spots (Figure 2C(i)) and diffused ring (Figure 2C(ii)) were identified to crystal planes and amorphous phase, respectively. The random atomic arrangement (inherent defects) could offer abundant active sites and electron channels, which promoted electron conduction and ion diffusion [27,28]. Apart from them, the high-angle annular dark-field scanning transmission electron microscope (HAADF-STEM) also indicated homogeneous distribution of C, Al, S, Co and N elements (Figure 2E), confirming C, Al and N species were well-introduced into the Co(Al)S matrix (according with SEM-EDS result in Figures S2 and S3).

**Figure 3:**
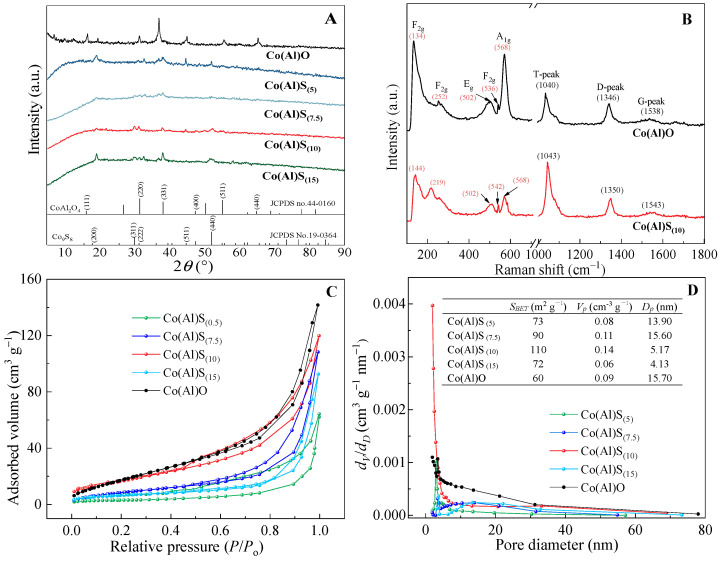
(**A**) XRD patterns of the Co(Al)O and the Co(Al)S; (**B**) Raman spectra of the Co(Al)O and the Co(Al)S_(10)_; (**C**) N_2_ adsorption–desorption isotherms; (**D**) pore size distributions of the Co(Al)O and the Co(Al)S.

The crystalline structures of the CAU-1, the Co(Al)O intermediate and the Co(Al)S were determined by XRD (Figure 3A and Figure S4). As illustrated in Figure S4, the diffraction peaks of the CAU-1 fitted well with the simulated crystallographic structure of the Al_4_(OH)_2_(OCH_3_)_4_(H_2_N-BDC)_3_ structure (generated using CIF file 723320, CAU-1), which was in accordance with those reported in the literature [29,30]. For the Co(Al)O intermediate, the six diffraction peaks at 16.2, 31.2, 36.7, 45.2, 59.2 and 65.0° were indexed to the (111), (220), (311), (400), (511) and (440) crystal planes of cubic spinel CoAl_2_O_4_ (JCPDS, no. 44-0160), accompanied by the vanishment of the characteristic peaks of the original CAU-1, suggesting that the self-sacrificial template CAU-1 was converted into CoAl_2_O_4_ after ion exchange/acid etching with Co(NO_3_)_2_. After the Co(Al)O was transformed into cobalt sulfide via sulfurization, the diffraction peaks at 18.3, 29.9, 31.2, 44.8 and 52.1° were assigned to the (200), (311), (222), (511) and (440) crystal planes of cubic Co_9_S_8_ (JCPDS, no. 19-0364) [31], ascertaining the successful conversion of the CoAl_2_O_4_ phase into the Co_9_S_8_ phase. The broadening and weakening peaks implied the amorphous/crystalline feature of the Co(Al)S [32], verifying the HRTEM observation. There was no obvious change in the XRD patterns for the Co(Al)S samples, suggesting that the amorphous/crystalline structure was not affected by the sulfur source concentration. Note that no signal about the phases containing C, N, and Al species emphasized that the three elements were introduced into the Co(Al)S matrix.

The crystallization of the Co(Al)S was also notarized by Raman, which Raman spectra of the Co(Al)O and the Co(Al)S_(10)_ are illustrated in Figure 3B. For the metal sulfides and cubic spinels, the Raman peaks at about 502 and 568 cm^−1^ could be indexed to the E_g_ and A_1g_ modes, respectively, while the peaks at around 132, 252 and 536 cm^−1^ belonged to the *F*_2g_ mode [33,34]. The Co(Al)S_(10)_ showed the characteristic peaks at 568 (A_1g_), 542 (F_2g_), 502 (E_g_), 219 (F_2g_) and 144 (F_2g_) cm^−1^ corresponding to the vibration modes of the Co_9_S_8_ phase [35]. The typical modes of cubic spinel CoAl_2_O_4_, A_1g_ (568 cm^−1^), F_2g_ (134, 252, 536 cm^−1^) and E_g_ (502 cm^−1^), were detected in the Co(Al)O intermediate [36]. Especially, the two peaks at around 1346 and 1538 cm^−1^ assigning to sp^3^ disordered carbon (D band, amorphous carbon) and sp^2^ graphite carbon (G band, graphitic structure) were observed in the Co(Al)O and the Co(Al)S_(10)_. The intensity ratio (*I*_D_/*I*_G_) of the D and G bands was relevant to the structural defect of materials. The *I*_D_/*I*_G_ values of the Co(Al)O and the Co(Al)S_(10)_ were 1.15 and 1.18, suggesting the existence of abundant structural defects (amorphous carbon) [37]. Additionally, the peak at around 1045 cm^−1^ was concerned with the T peak due to the stretch mode of the N-C bond [38], revealing the existence of amorphous C and N species. The Raman results finalized the deduction from the XRD and the TEM/HRTEM that amorphous C, N and Al species as were introduced into the Co(Al)S matrix.

The textural properties such as pore volume (*V*_P_), pore diameter (*D*_P_) and specific surface area (*S*_BET_) were investigated by N_2_ absorptiometry (Figure 3C). All the N_2_ adsorption–desorption isotherms presented the type-IV isotherm with H3 hysteresis loops due to the aggregates of nanosheets [39], and the corresponding pore size distributions were in the range of 2.0~35 nm, validating the mesoporous layered structure (Figure 3D). Obviously, the *S*_BET_ of the Co(Al)S was larger than that of the Co(Al)O intermediate (Figure 3D inset), and the Co(Al)S_(10)_ exhibited the largest *S*_BET_ (110 m^2^ g^–1^), due to the 3D network architecture composed of individually ultrathin nanosheets via sulfuration in a moderate concentration of sulfur source. High *S*_BET_ could expose more active sites on the surface of materials to accelerate electrons/ions transfer and Faradaic reaction kinetics.

**Figure 4:**
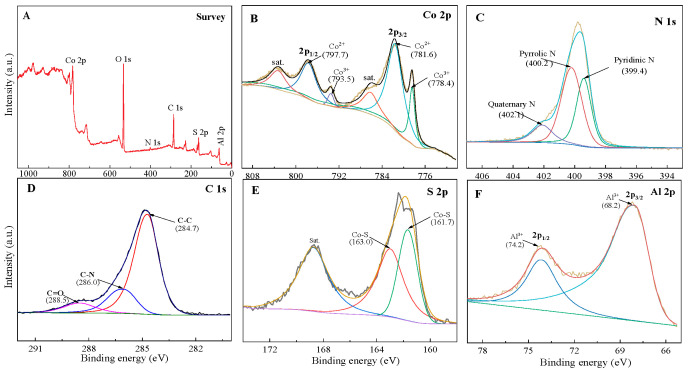
XPS spectrum of the Co(Al)S_(10)_ (survey (**A**), Co 2p (**B**), N 1s (**C**), C 1s (**D**), S 2p (**E**) and Al 2p (**F**)).

The surface chemical state and composition of the Co(Al)S_(10)_ were clarified by XPS, as shown in Figure 4. The XPS survey spectrum revealed Co, O, N, C, Al and S elements (Figure 4A), which coincided with the SEM-EDS and HAADF-STEM results. The O element came from adventitious O species, which was confirmed by O 1s spectrum (Figure S5) and C 1s spectra (Figure 4D). The peaks at 288.5 and 284.7 eV were attributed to the O-C=O mode of absorbed CO_2_ from air [40] and the C-C bond of adventitious carbon as calibration references [41], respectively, while the peak at 286.0 eV was ascribed to the C-N bond [42]. Additionally, the N 1s spectrum (Figure 4C) displayed the peaks assigning to pyridinic N (399.4 eV), pyrrolic N (400.2 eV) and quaternary N (402.1 eV) [43], ensuring the C and N species in the Co(Al)S matrix. The Co 2p XPS spectrum (Figure 4B) was composed of two spin-orbit doublets (2p_1/2_ and 2p_3/2_) and two shake-up satellites (denoted as Sat.), where the fitting peaks at 797.7 and 781.6 eV were ascribed to the Co^2+^ state, and the other peaks at 793.5 and 778.4 eV were the typical Co^3+^ state [39,41]. In the S 2p spectrum (Figure 4E), the peaks at around 163.0 and 161.7 eV were related to metal–sulfur bonds (Co-S) from S^2−^ [39]. The Al 2p XPS showed two peaks at 74.2 eV (Al 2p_1/2_) and 68.2 eV (Al 2p_3/2_) due to coordination of Al^3+^ [39,44] (Figure 4F).

### 3.3. Electrochemical Performance of Materials

**Figure 5:**
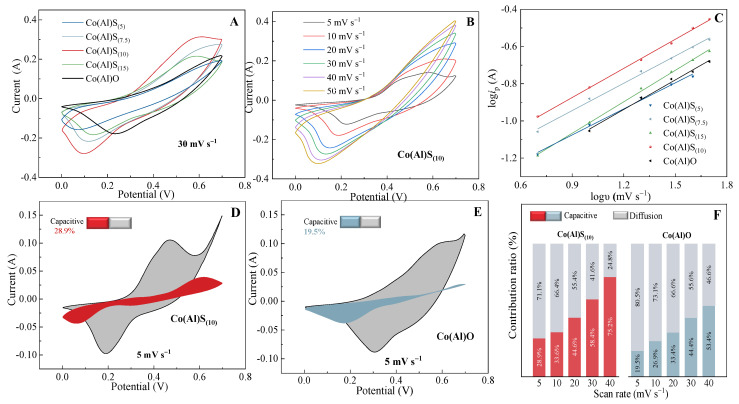
(**A**) CV curves of the Co(Al)O and the Co(Al)S at 30 mV s^–1^; (**B**) CV curves of the Co(Al)S_(10)_ at different scan rates; (**C**) plots of log*i* vs. log*υ* for the CoAl_2_O_4_ and the Co(Al)S; (**D**) capacitive contribution of the Co(Al)S_(10)_ and the Co(Al)O (**E**) at 5 mV s^–1^; (**F**) histograms of the capacitance contributions for the Co(Al)S_(10)_ (red area) and the Co(Al)O (blue-gray area) at different scan rates.

**Figure 6:**
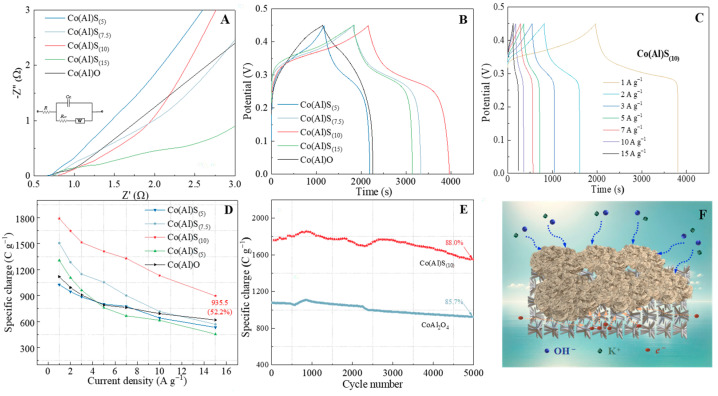
Nyquist plots (**A**) and GCD profiles at 1 A g^−1^ (**B**) of the Co(Al)O and the Co(Al)S; GCD profiles of the Co(Al)S_(10)_ at different current densities (**C**); specific charges of the Co(Al)O and the Co(Al)S at different current densities (**D**); cycling stability of the Co(Al)O and the Co(Al)S_(10)_ (**E**); schematic illustration of the energy storage mechanism of the Co(Al)S (**F**).

**Figure 7:**
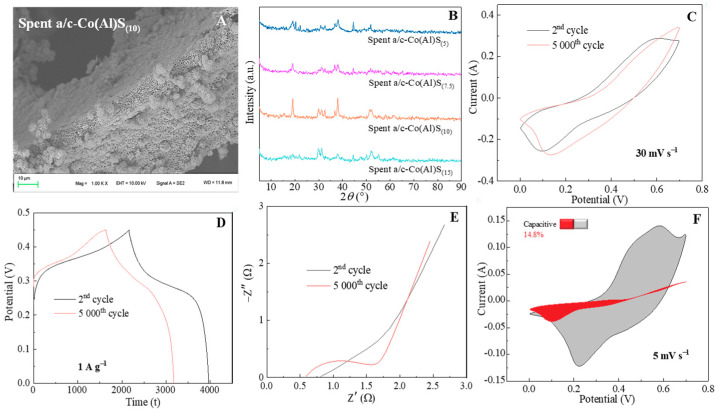
SEM(**A**) and XRD images (**B**) of the spent Co(Al)S_(10)_; CV curves at 30 mV s^–1^ (**C**); GCD profiles at 1 A g^–1^ (**D**); and Nyquist plots (**E**) of the Co(Al)S_(10)_ before and after 5000 cycles. Capacitance contribution (**F**) of the spent Co(Al)S_(10)_ at 5 mV s^−1^.

The electrochemical properties of the Co(Al)O intermediate and the Co(Al)S as electrode materials were evaluated by CV, EIS and GCD in a three-electrode system (Figure 5, Figure 6, Figure 7 and Figures S6–S8). Firstly, the CV curves at the 12th cycle of the Co(Al)O and the Co(Al)S at 30 mV s^−1^ are displayed in Figure 5A. Clearly, the CV curves showed a pair of distinct redox peaks, suggesting the typical Faradaic reaction feature [41]. The Co(Al)S_(10)_ demonstrated the strongest peak current and the largest CV curve integrated area (*A*_area_, 12.21 × 10^−2^) (Table S1), implying the highest specific charge (*C*s) and electrochemical reaction activity. In order to clarify the charge storage mechanism and the reaction kinetics of the Co(Al)S, the CV behavior of the samples was evaluated at various scan rates, in which there was little change in the shape of the CV curves from 5 to 50 mV s^−1^ (Figure 5B and Figure S6), showing good reversibility and rate capability [39]. Furthermore, the reaction kinetics were studied by quantitatively analyzing the relationship between response current (*i*, mA) and scan rate (*υ*, mV s^−1^) based on the equation: *I* = *aυ^b^* (*a* and *b* are the adjustable parameters). The *b*-value comes out to the slope of log*i* vs. log*υ*, in which *b*-values close to 0.5 and 1 present a diffusion process (battery-type behavior) and a surface-controlled process (capacitive-type behavior) [39,41], respectively. In our case, the *b*-values (0.47~0.56) were close to 0.5 (Figure 5C and Table S1), proving that the redox reactions in the Co(Al)O and the Co(Al)S were controlled by ion diffusion (typical battery-type behavior). Additionally, the good linear relationship of the square root of the scan rate (*υ*^1/2^) vs. anodic peak currents (*i*_pc_) ascertained primarily diffusion-limited redox reactions (Figure S7 and Table S1). So, the capacitive (*k*_1_*υ*, fast reaction kinetics) and diffusive (*k*_2_*υ*^1/2^, sluggish reaction kinetics) contributions to total charge storage were quantified by the following Equation (1) [41,42],

*i* = *k*_1_*υ* + *k*_2_*υ*^1/2^ (1)

where *i* and *υ* are the response current (mA) at a fixed potential (V) and the scan rate (mV s^−1^), respectively. The *k*_1_ and *k*_2_ constants are the slope and the intercept of the plot by a linear fit between *i*/*υ*^1/2^ and *υ*^1/2^ at a specific scan rate [41]. As shown in Figure 5D,E, the capacitance contributions of the Co(Al)S_(10)_ and the Co(Al)O were 28.9% (red region) and 19.5% (blue-gray region) at 5 mV·s^−1^, uncovering that diffusion-controlled capacity occupied a dominating portion of the total charge storage [41,45,46]. The higher capacitive contribution meant a faster charge transfer [27], revealing the Co(Al)S_(10)_ had a higher conductivity and ion accessibility than the Co(Al)O due to higher surface area. Meanwhile, the capacitive contribution gradually increased with quickening the scan rate (Figure 5F), where the diffusion process was suppressed and the surface-controlled process became dominant due to inadequate redox reaction time at high scan rates. Thus, the charge storage was converted into a capacitive-type capacity dominance.

The reaction kinetic properties of the Co(Al)S were also evaluated by EIS, and the related Nyquist plots are presented in Figure 6A. The plots indicated a semicircle relative to the charge transfer resistance (*R*_ct_) in the high-frequency region and a line in the low-frequency region (diffusion limited process) [27,46]. The *R*_ct_ and *R*_s_ (equivalent series resistance) values were calculated by fitting the EIS data to the suitable equivalent circuit (chi-square values ≤ 2.23 × 10^−3^) (Figure 6A inset and Table S1). As expected, the Co(Al)S_(10)_ displayed the lowest *R*_ct_ value (0.13 Ω), determining the strongest electron transfer capability.

To evaluate charge storage capacity, GCD performances of the Co(Al)O intermediate and the Co(Al)S were investigated at the second cycle (Figure 6B–E and Figure S8). As seen in Figure 6B, the Faradaic battery-type behavior and the good reversibility were further manifested by almost symmetric and nonlinear GCD curves [39,41,45]. The Co(Al)S_(10)_ displayed the longest discharging time and the highest specific charge (*C*s, 1791.8 C g^−1^) at 1 A g^−1^ (Table S1), which was consistent with the CV result. The GCD curves of the Co(Al)O and the Co(Al)S at different current densities are also presented in Figure 6C and Figure S8, where the *C*s decreased with enhancing current density due to insufficient diffusion rate at higher current density. The specific charge of the Co(Al)S_(10)_ was higher than those of the Co(Al)O intermediate and the other Co(Al)S materials at each current density (Figure 6D), highlighting the excellent overall electrochemical performance. Furthermore, the specific charges of the Co(Al)S_(10)_ were 1791.0, 1646.2, 1513.2, 1409.5, 1329.6, 1130.0 and 935.5 C g^−1^ at 1, 2, 3, 5, 7, 10 and 15 A g^−1^, respectively. Though the rate property of the Co(Al)S was lower than that of the Co(Al)O, the Co(Al)S_(10)_ still had 52.2% retention up to 15.0 A g^−1^ (Table S1), confirming good rate capability. Moreover, the long-term cycle stabilities of the Co(Al)O and the Co(Al)S_(10)_ were evaluated by repeating the GCD at 1 A g^−1^ for 5000 cycles (Figure 6E). The Co(Al)S_(10)_ still kept a high specific charge of 1549.5 C g^−1^ (88.0% retention) with 98.4% coulomb efficiency after 5000 cycles, better than the CoAl_2_O_4_ (85.7% retention) with 99.0% coulomb efficiency, suggesting excellent cycling stability. The electrochemical test results revealed that suitable sulfuration endowed materials with rapid ion/electron transport, higher electrical conductivity and excellent specific capacity, thus leading to enhancement in electrochemical performance.

In order to further evaluate the structural stability in the cycling process, the spent Co(Al)S_(10)_ after 5000 cycles was evaluated by SEM, XRD, CV, GCD and EIS (Figure 7). The spent Co(Al)S_(10)_ kept the initial morphology with a 3D flower-like “sand-rose” structure (Figure 7A), indicating good structural stability. It was found that the peak intensities increased and the baseline of the XRD traces became stable after 5000 cycles (Figure 7B), meaning increased crystallinity. The enhanced crystallinity was attributed to transformation of the unstable amorphous phase in the Co(Al)S into the crystalline Co_9_S_8_ phase in the charge–discharge process [47]. There was a similar shape in the CV and the GCD curves recorded for the Co(Al)S_(10)_ at the 2nd and 5000th cycles, though the CV curve integrated area and the specific charge (1576.1 C g^−1^) slightly declined (Figure 7C,D). As illustrated in Figure 7E,F, the Co(Al)S_(10)_ exhibited an increased *R*_ct_ value (0.925 Ω), and a declined capacitive contribution (14.8%), revealing depressed charge transport and Faradaic reaction kinetics. The descent in the electrochemical properties was mainly due to transformation of the amorphous into the crystalline phase in the Co(Al)S and aggregation of nanosheets. The results showed that the Co(Al)S_(10)_ possessed high structure and cycling stability.

Taking the above structural characterization and electrochemical properties together, this could come to the conclusion that there is viability in the Co(Al)S heterostructure via the MOF-derived synthesis strategy, in which the Co(Al)O intermediate was obtained via ion exchange/acid etching the CAU-1 self-sacrificial template and then sulfurized into the Co(Al)S with Al, N and C coordinated amorphous/crystalline heterophase structure. The optimal Co(Al)S_(10)_ obtained by adjusting the sulfur source concentration to a suitable level exhibited excellent specific charge, rapid charge transfer and cycling and structural stability, which was superior or comparable to the previously reported transition metal sulfides on NF (Table S2). The enhanced electrochemical performance was attributed to collaboration of the unique amorphous/crystalline heterophase structure and the 3D nanosheet-interconnected network architecture, as schematically shown in Figure 6. (1) The amorphous/crystalline heterophase could give rich interfaces and expose abundant active sites to accelerate Faradaic redox reaction. (2) The N and C species coordinated in the amorphous phase boosted electron transport and ion diffusion. (3) The 3D ultrathin-nanosheet-interlaced structure provided more electron/ion ‘‘superhighways”, improving efficient charge delivery and storage. (4) Current collector NF as the direct support endowed the bind-free structure to promote ion/electron transport and avoided aggregation of nanosheets to ensure the stability of cycling and structure. As a result, the Co(Al)S was a competitive candidate as electrode material for supercapacitors.

### 3.4. Performance of the Co(Al)S//AC Hybrid Supercapacitor

**Figure 8:**
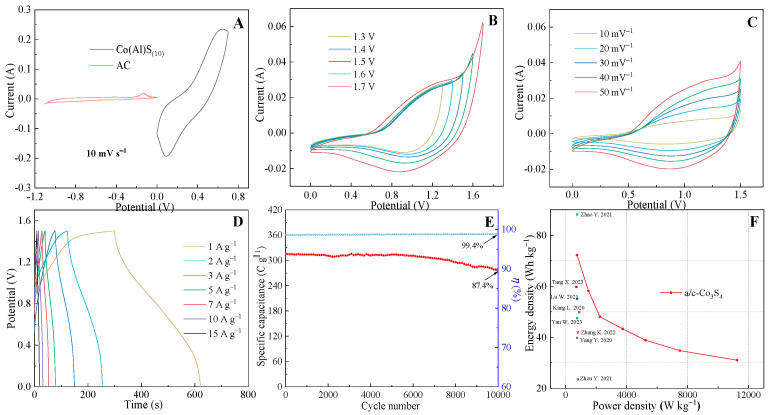
Supercapacitor performances of the Co(Al)S//AC device. (**A**) CV curves of the AC and the Co(Al)S_(10)_ at 10 mV s^−1^ in a three-electrode system; (**B**) CV curves at 10 mV s^−1^ in different working potentials; (**C**) CV curves at 1.5 V in different scan rates; (**D**) GCD curves at different current densities; (**E**) cycling stability at 1.0 A g^−1^; (**F**) Ragone plots [14,24,27,39,46,48,49].

To evaluate potential application of the Co(Al)S, a hybrid supercapacitor (HSC, Co(Al)S//AC) was fabricated using the Co(Al)S_(10)_ and activated carbon (AC) as positive and negative electrodes (Figure 8). At 10 mV s^−1^ in a three-electrode system, the stable potential windows of the negative CV (−1.0~0 V) and the positive Co(Al)S_(10)_ (0~0.7 V) are displayed in Figure 8A, showing that the working voltage of the HSC device could be enlarged to 1.7 V. As seen in Figure 8B, the CV curve started to be polarized at 1.6 V at 50 mV·s^−1^, suggesting that the optimal operating voltage was 1.5 V. A sequence of CV curves at 1.5 V illustrated the feature of both battery-type in the high voltage region and capacitive-type storage in the low voltage region, showing the combination of redox reaction and physical adsorption/desorption process (Figure 8C). At the same time, there was no obvious change at various scan rates, indicating good rate capability. The quasi-trigonal GCD curves proved the mixed charge storage mechanism and a good match between the anode and cathode materials (Figure 8D). The specific capacitances of the device were calculated to be 325.1, 261.9, 246.3, 214.8, 199.9, 184.4 and 168.8 C g^−1^ at current densities of 1, 2, 3, 5, 7, 10 and 15 A g^−1^, respectively. Therefore, the HSC device maintained 51.9% rate property even up to a 15-times increment of the current density. The cycling stability of the Co(Al)S//AC device was also studied at 1 A g^−1^ under 1.5 V, in which the device still kept 87.4% of its initial specific capacitance with 99.8% columbic efficiency for 10,000 cycles, showing an excellent cycling stability (Figure 8E). Furthermore, the HSC device brought an energy density of 72.3 Wh kg^−1^ at a power density of 750 W kg^−1^ and still remained 31.1 Wh kg^−1^ at 11,250 W kg^−1^, which was comparable or even higher than those of the previously reported HSC devices using Ni/Co-based sulfides as the positive electrode (Figure 8F and Table 1). This demonstrated that the as-fabricated Co(Al)S//AC device would be a potential candidate for supercapacitor applications.

## 4. Conclusions

A novel self-templated MOF strategy was employed to construct cobalt sulfide with aluminum, nitrogen and carbon coordinated amorphous/crystalline heterophase (denoted as Co(Al)S), where the Co(Al)S nanosheets were synthesized by ion exchange/acid etching the CAU-1 on NF followed by sulfuration. Rational sulfidation by adjusting the sulfur source to a suitable concentration level helped form a 3D nanosheet-interconnected network architecture with a high specific surface area. The features of the unique 3D structure network and crystalline/amorphous coordinated Al, N, C atoms could shorten ion diffusion paths and expose affluent active sites to accelerate electron transport and ion diffusion, leading to excellent electrochemical properties. As a result, the perfected Co(Al)S_(10)_ exhibited a high specific charge of 1791.8 C g^−1^ at 1 A g^−1^, a satisfying rate property (52.2% retention at 15 A g^−1^) and a good cycling stability (88.0% retention at 1 A g^−1^ after 5000 cycles). Furthermore, the assembled Co(Al)S//AC device achieved an energy density of 72.3 Wh kg^−1^ at a power density of 750 W kg^−1^, with an excellent cyclic reliability of 87.4% at 1 A g^−1^ after 10,000 cycles. This work provides a feasible strategy for designing and constructing high-performance MOF-derived electrode materials in energy-related applications.

## Figures and Tables

**Table 1:** Comparison of the Co(Al)S//AC device with the reported Ni/Co-sulfide-based HSC devices.

	KOH Electrolyte (mol L^−1^)	Working Voltage (V)	Energy Density (Wh kg^−1^)	Power Density (W kg^−1^)	Cycling Stability	Ref.
Ni-Ni_3_S_4_/CNTs//CNTs *	3	1.5	39.8	749.8	82.8% (15,000 cycles at 1 A g^−1^)	[14]
NCM-S@HCNS//N-rGO *	2	1.6	23.5	800.4	87.7% (10,000 cycles at 10 A g^−1^)	[25]
NiCoS//porous carbon	6	1.6	59.7	710	92.3% (10,000 cycles at 5 A g^−1^)	[27]
NiCoMoS@NiCoAl-LDH//AC	3	1.6	41.9	800	89.3% (10,000 cycles at 5 A g^−1^)	[39]
NiCoMnS//AC	3	1.5	47.5	750.0	85.2% (10,000 cycles at 5 A g^−1^)	[41]
FC@MS-EG *//AC	6	1.5	88.1	750.0	84.7% (6000 cycles at 8 A g^−1^)	[46]
FM-Co_3_S_4_ *//AC	6	1.5	55.0	752.0	86.1% (5000 cycles at 10 A g^−1^)	[48]
F-CuCo_2_S_4_//AC	1	1.6	49.8	897.4	89.2% (10,000 cycles at 20 A g^−1^)	[49]
Co(Al)S//AC	3	1.6	72.3	750	87.5% (10,000 cycles at 1 A g^−1^)	This work

*: CNTs: carbon nanotubes; NCM-S: Ni-Co-S/crystalline MnS; HCNS: hollow carbon nanospheres; N-rGO N-doped reduced graphene oxide; FC: FeCo-based nanoneedles; MS-EG: MoS_2_ prepared in absolute ethanol; FM-Co_3_S_4_: Fe and Mn co-doped Co_3_S_4_.

## Data Availability

The original contributions presented in the study are included in the article/Supplementary Material.

## Supplementary Materials

The following supporting information can be downloaded at: https://www.mdpi.com/article/10.3390/ma17164030/s1, Figure S1: Distribution of nanosheet thickness for the Co(Al)S based the SEM images; Figure S2: SEM-EDS pattern of the Co(Al)S(10) sample; Figure S3: SEM and EDS-mapping images as well as the corresponding element distributions of the Co(Al)S(10) sample; Figure S4: XRD patterns of the CAU-1 self-sacrificial template; Figure S5: High-resolution O 1 s XPS spectrum of the Co(Al)S(10); Figure S6: CV curves of the Co(Al)O, Co(Al)S(5), Co(Al)S(7.5) and Co(Al)S(15) at different scan rates; Figure S7: Randles-Sevcik plots of the peak current vs. square root of scan rate for the samples; Figure S8: GCD profiles of the Co(Al)O, Co(Al)S(5), Co(Al)S(7.5) and Co(Al)S(15) at different current densities; Table S1: Electrochemistry performances of the Co(Al)O and Co(Al)S; Table S2: Electrochemical properties of the Co(Al)S compared to the reported transition metal sulfides in the three-electrode system. References [12,14,17,21,25,27,39,41,45,46,48,50,51] are cited in Supplementary Materials.

## References

[1] Ali Z., Iqbal M.Z., Hegazy H.H. (2023). Recent advancements in redox-active transition metal sulfides as battery-grade electrode materials for hybrid supercapacitors. J. Energy Storage.

[2] Park H.W., Roh K.C. (2023). Recent advances in and perspectives on pseudocapacitive materials for supercapacitors—A review. J. Power Sources.

[3] Dong W., Xie M., Zhao S., Qin Q., Huang F. (2023). Materials design and preparation for high energy density and high power density electrochemical supercapacitors. Mater. Sci. Eng. R.

[4] Liu Y., Xu X., Shao Z. (2020). Metal-organic frameworks derived porous carbon, metal oxides and metal sulfides-based compounds for supercapacitors application. Energy Storage Mater..

[5] Mustaqeem M., Naikoo G.A., Yarmohammadi M., Pedram M.Z., Pourfarzad H., Dar R.A., Taha S.A., Hassan I.U., Bhat M.Y., Chen Y.F. (2022). Rational design of metal oxide-based electrode materials for high performance supercapacitors—A review. J. Energy Storage.

[6] Li X., Ren J., Sridhar D., Xu B.B., Algadi H., El-Bahy Z.M., Ma Y., Li T., Guo Z. (2023). Progress of layered double hydroxide-based materials for supercapacitors. Mater. Chem. Front..

[7] Tang G., Liang J., Wu W. (2024). Transition metal selenides for supercapacitors. Adv. Funct. Mater..

[8] Öztürk O., Gür E. (2024). Layered transition metal sulfides for supercapacitor applications. ChemElectroChem.

[9] Gao Y., Zhao L. (2022). Review on recent advances in nanostructured transition-metal-sulfide-based electrode materials for cathode materials of asymmetric supercapacitors. Chem. Eng. J..

[10] Dahiya Y., Hariram M., Kumar M., Jain A., Sarkar D. (2022). Modified transition metal chalcogenides for high performance supercapacitors: Current trends and emerging opportunities. Coordin. Chem. Rev..

[11] Theerthagiri J., Senthil R.A., Nithyadharseni P., Lee S.J., Durai G., Kuppusami P., Madhavan J., Choi M.Y. (2020). Recent progress and emerging challenges of transition metal sulfides based composite electrodes for electrochemical supercapacitive energy storage. Ceram. Int..

[12] Hao C., Ni C., Wang X., Pan Y., Wu Q., Wu J., Wang X. (2023). Fabrication of three-dimensional CuS_2_@CoNi_2_S_4_ core-shell rod-like structures as cathode and thistle-derived carbon as anode for hybrid supercapacitors. Chem. Eng. J..

[13] Shi Y., Zhu B., Guo X., Li W., Ma W., Wu X., Pang H. (2022). MOF-derived metal sulfides for electrochemical energy applications. Energy Storage Mater..

[14] Yang Y., Li M.L., Lin J.N., Zou M.Y., Gu S.T., Hong X.J., Si L.P., Cai Y.P. (2020). MOF-derived Ni_3_S_4_ encapsulated in 3D conductive network for high-performance supercapacitor. Inorg. Chem..

[15] Peng Y., Bai Y., Liu C., Cao S., Kong Q., Pang H. (2022). Applications of metal-organic framework-derived N, P, S doped materials in electrochemical energy conversion and storage. Coordin. Chem. Rev..

[16] Nagaraju G., Sekhar S.C., Ramulu B., Yu J.S. (2021). High-performance hybrid supercapacitors based on MOF-derived hollow ternary chalcogenides. Energy Storage Mater..

[17] Kang C., Ma L., Chen Y., Fu L., Hu Q., Zhou C., Liu Q. (2022). Metal-organic framework derived hollow rod-like NiCoMn ternary metal sulfide for high-performance asymmetric supercapacitors. Chem. Eng. J..

[18] Huang C., Gao A., Yi F., Wang Y., Shu D., Liang Y., Zhu Z., Ling J., Hao J. (2021). Metal organic framework derived hollow NiS@C with S-vacancies to boost high-performance supercapacitors. Chem. Eng. J..

[19] Chen J., Sun X., Kong W., Yu Q., Long Y., Zhou T., Hu C., Dai Y., Gong J., Pu L. (2023). N-doped bimetallic sulfides hollow spheres derived from MOF as battery-type electrode for asymmetric supercapacitors. J. Energy Storage.

[20] Zhang Y., Gao F., Wang D., Li Z., Wang X., Wang C., Zhang K., Du Y. (2023). Amorphous/crystalline heterostructure transition-metal-based catalysts for high-performance water splitting. Coordin. Chem. Rev..

[21] Huang B., Yuan J., Lu Y., Zhao Y., Qian X., Xu H., He G., Chen H. (2022). Hollow nanospheres comprising amorphous NiMoS_4_ and crystalline NiS_2_ for all-solid-state supercapacitors. Chem. Eng. J..

[22] Jin Y., Zhang M., Song L., Zhang M. (2023). Research advances in amorphous-crystalline heterostructures toward efficient electrochemical applications. Small.

[23] Gao D., Pan Y., Wei J., Han D., Xu P., Wei Y., Mao L., Yin X. (2022). Interfacial engineering in amorphous/crystalline heterogeneous nanostructures as a highly effective battery-type electrode for hybrid supercapacitors. J. Mater. Chem. A.

[24] Chao D., DeBlock R., Lai C.H., Wei Q., Dunn B., Fan H.J. (2021). Amorphous VO_2_: A pseudocapacitive platform for high-rate symmetric batteries. Adv. Mater..

[25] Zhou Y., Jia Z., Zhao S., Chen P., Wang Y., Guo T., Wei L., Cui X., Ouyang X., Wang X. (2021). Construction of triple-shelled hollow nanostructure by confining amorphous Ni-Co-S/crystalline MnS on/in hollow carbon nanospheres for all-solid-state hybrid supercapacitors. Chem. Eng. J..

[26] Xue W.L., Deng W.H., Chen H., Liu R.H., Taylor J.M., Li Y.K., Wang L., Deng Y.H., Li W.H., Wen Y.Y. (2021). MOF-directed synthesis of crystalline ionic liquids with enhanced proton conduction. Angew. Chem. Int. Ed..

[27] Tang X., Wang J., Zhang D., Wang B., Xia X., Meng X., Yang B., Chen J., He Y., Han Z. (2023). In-situ construction of carbon cloth-supported amorphous/crystalline hybrid NiCo-sulfide with permeable concrete-like morphology for high-performance solid-state hybrid supercapacitors. Chem. Eng. J..

[28] Wang Z., Song Y., Wang J., Lin Y., Meng J., Cui W., Liu X.X. (2023). Vanadium oxides with amorphous-crystalline heterointerface network for aqueous zinc-ion batteries. Angew. Chem. Int. Ed..

[29] Zheng X., Liu S., Rehman S., Li Z., Zhang P. (2020). Highly improved adsorption performance of metal-organic frameworks CAU-1 for trace toluene in humid air via sequential internal and external surface modification. Chem. Eng. J..

[30] Gumber N., Pai R.V., Bahadur J., Sengupta S., Das D., Goutam U.K. (2023). γ-Resistant microporous CAU-1 MOF for selective remediation of thorium. ACS Omega.

[31] Zhao Y., Pang Q., Wei Y., Wei L., Ju Y., Zou B., Gao Y., Chen G. (2017). Co_9_S_8_/Co as a high-performance anode for sodium-ion batteries with an ether-based electrolyte. ChemSusChem.

[32] Cheng Q., Yang Z., Li Y., Wang J., Wang J., Zhang G. (2023). Amorphous/crystalline Cu_1.5_Mn_1.5_O_4_ with rich oxygen vacancies for efficiently photothermocatalytic mineralization of toluene. Chem. Eng. J..

[33] Zeng P., Li J., Ye M., Zhuo K., Fang Z. (2017). In situ formation of Co_9_S_8_/N-C hollow nanospheres by pyrolysis and sulfurization of ZIF-67 for high-performance lithium-ion batteries. Chem. Eur. J..

[34] Álvarez-Docio C.M., Reinosa J.J., Del Campo A., Fernández J.F. (2019). Investigation of thermal stability of 2D and 3D CoAl_2_O_4_ particles in core-shell nanostructures by Raman spectroscopy. J. Alloy. Compd..

[35] Mujtaba J., He L., Zhu H., Xiao Z., Huang G., Solovev A.A., Mei Y. (2021). Co_9_S_8_ nanoparticles for hydrogen evolution. ACS Appl. Nano Mater..

[36] Li Y., Zhao Z., Zhao M., Zhu H., Ma X., Li Z., Lu W., Chen X., Ying L., Lin R. (2024). Oxygen-vacancy induced structural changes of Co species in CoAl_2_O_4_ spinels for CO_2_ hydrogenation. Appl. Catal. B Environ. Energ..

[37] Zou J., Lin Y., Wu S., Zhong Y., Yang C. (2021). Molybdenum dioxide nanoparticles anchored on nitrogen-doped carbon nanotubes as oxidative desulfurization catalysts: Role of electron transfer in activity and reusability. Adv. Funct. Mater..

[38] Cheng Y.H., Tay B.K., Lau S.P., Shi X., Qiao X.L., Sun Z.H., Chen J.G., Wu Y.P., Xie C.S. (2001). Influence of nitrogen ion energy on the Raman spectroscopy of carbon nitride films. Diam. Relat. Mater..

[39] Zhang K., Zeng H.Y., Wang M.X., Li H.B., Yan W., Wang H.B., Tang Z.H. (2022). 3D hierarchical core-shell structural NiCoMoS@NiCoAl hydrotalcite for high-performance supercapacitors. J. Mater. Chem. A.

[40] Jiang Z., Zhang M., Chen X., Wang B., Fan W., Yang C., Yang X., Zhang Z., Yang X., Li C. (2023). A bismuth-based zeolitic organic framework with coordination-linked metal cages for efficient electrocatalytic CO_2_ reduction to HCOOH. Angew. Chem. Int. Ed..

[41] Yan W., Zeng H.Y., Zhang K., Long Y.W., Wang M.X. (2023). Ni-Co-Mn hydrotalcite-derived hierarchically porous sulfide for hybrid supercapacitors. J. Colloid Interf. Sci..

[42] Kumar M., Jeong D.I., Sarwar N., Dutta S., Chauhan N., Han S.A., Kim H.J., Yoon D.H. (2022). Cobalt supported nitrogen-doped carbon nanotube as efficient catalyst for hydrogen evolution reaction and reduction of 4-nitrophenol. Appl. Surf. Sci..

[43] Yang Z., Wang J., Wu H.T., Kong F.J., Yin W.Y., Cheng H.J., Tang X.Y., Qian B., Tao S., Yi J. (2019). MOFs derived Co_1-x_S nanoparticles embedded in N-doped carbon nanosheets with improved electrochemical performance for lithium ion batteries. Appl. Surf. Sci..

[44] Gu S., Wang H., Wu C., Bai Y., Li H., Wu F. (2017). Confirming reversible Al^3+^ storage mechanism through intercalation of Al^3+^ into V_2_O_5_ nanowires in a rechargeable aluminum battery. Energy Storage Mater..

[45] Tang Z.H., Zeng H.Y., Zhang K., Yue H.L., Tang L.Q., Lv S.B., Wang H.B. (2024). Engineering core-shell NiC_2_O_4_@C/N-direct-doped NiCoZn-LDH for supercapacitors. Chem. Eng. Sci..

[46] Zhao Y., Wang S., Yuan M., Chen Y., Huang Y., Lian J., Yang S., Li H., Wu L. (2021). Amorphous MoS_x_ nanoparticles grown on cobalt-iron-based needle-like array for high-performance flexible asymmetric supercapacitor. Chem. Eng. J..

[47] Guo J., He B., Gong W., Xu S., Xue P., Li C., Sun Y., Wang C., Wei L., Zhang Q. (2024). Emerging amorphous to crystalline conversion chemistry in Ca-doped VO_2_ cathodes for high-capacity and long-term wearable aqueous zinc-ion batteries. Adv. Mater..

[48] Lu W., Yang Y., Zhang T., Ma L., Luo X., Huang C., Ning J., Zhong Y., Hu Y. (2021). Synergistic effects of Fe and Mn dual-doping in Co_3_S_4_ ultrathin nanosheets for high-performance hybrid supercapacitors. J. Colloid Interf. Sci..

[49] Kang L., Huang C., Zhang J., Zhang M., Zhang N., Liu S., Ye Y., Luo C., Gong Z., Wang C. (2020). Effect of fluorine doping and sulfur vacancies of CuCo_2_S_4_ on its electrochemical performance in supercapacitors. Chem. Eng. J..

[50] Ferrah D., Haines A.R., Galhenage R.P., Bruce J.P., Babore A.D., Hunt A., Waluyo I., Hemminger J.C. (2019). Wet chemical growth and thermocatalytic activity of Cu-based nanoparticles supported on TiO_2_ nanoparticles/HOPG: In situ ambient pressure XPS study of the CO_2_ hydrogenation reaction. ACS Catal..

[51] Burghaus U. (2014). Surface chemistry of CO_2_-Adsorption of carbon dioxide on clean surfaces at ultrahigh vacuum. Prog. Surf. Sci..

