# Supplementary material for: In-Situ Sulfuration of CoAl Metal–Organic Framework for Enhanced Supercapacitor Properties
Source: Materials (Basel). 2024 Aug 13;17(16):4030. doi: 10.3390/ma17164030 (PMC11356338; doi:10.3390/ma17164030)
Supplement: Supplementary file 1 [file materials-17-04030-s001.zip › materials-3152766-supplementary.pdf]

Supporting materials

In-Situ Sulfuration of CoAl Metal–Organic Framework for Enhanced Supercapacitor Properties

Mengchen Liao[1]*, Kai Zhang[2,3], Chaowei Luo[3], Guozhong Wu[2], Hongyan Zeng[3]

1. *School of Chemistry and Chemical Engineering, Central South University, Changsha, 410083, Hunan, China*

2. *Shanghai Institute of Applied Physics, Chinese Academy of Sciences, Shanghai 201800, China*

3. *College of Chemical Engineering, Xiangtan University, Xiangtan, 411105, Hunan, China*

*Corresponding author

Mengchen Liao

E-mail address: liaom6@csu.edu.cn

Mengchen Liao and Kai Zhang contributed equally to this work.

Catalogue

Experimental........1

Characterization........1

Electrochemical measurements........1

Figure S1........4

Figure S2........5

Figure S3........6

Figure S4........7

Figure S5........8

Figure S6........9

Figure S7........10

Figure S8........11

Table S1........12

Table S2........13

Experimental

Characterization

The samples were stripped from the NF by ultrasonic vibration and collected for XRD, Raman, TEM, EDS, N_2 sorptometry and XPS analyses. The phase structure was characterized by powder X-ray diffraction (XRD, Rigaku Rint-2000, Cu Kα). The structural characteristics were identified by Raman microscopy (Witec alpha 300 R) with 532 nm laser. The specific surface area and pore distribution were obtained by N_2 adsorptometry (Quantachrome NOVA-e1000) at −196°C. The morphology, structure and composition were measured using scanning electron microscopy with energy dispersive spectrometer (SEM/EDS, SU5000) and transmission electron microscopy coupled with energy dispersive Xray spectroscopy (TEM/EDS, JEOL JEM2100). Based the SEM images, the average thickness of the nanosheets was estimated using the Gaussian Fitting with ImageJ software. The chemical composition and state were determined using X-ray photoelectron spectroscopy (XPS, ULVAC-PHI PHI X-tool), in which the C1s peak from the adventitious carbon-based contaminant with a binding energy of 284.8 eV was used as the reference for calibration.

Electrochemical measurements

The electrochemical performance of the as-prepared samples was evaluated in three-electrode system on a CHI660E electrochemical workstation (Shanghai Chenhua Instruments Co., Ltd., China) with 3.0 mol L^{-1} KOH solution as the electrolyte, in which Hg/HgO electrode, platinum sheet (1×1 cm^2) and as-prepared materials were as the reference, counter and working electrodes, respectively. The cyclic voltammetry (CV) curves were collected between −0.3 V and 0.9 V at the 12th cycle, and galvanostatic charge-discharge (GCD) conducted between 0 and 0.45 V, while the electrochemical impedance spectroscopy (EIS) was examined in the frequency range from 0.01 Hz to 100 kHz under open circuit potential with 5 mV amplitude. The specific charge (C_s) and coulomb efficiency (η) were calculated using Eqs. (S1) and (S2) [1],

$$C_S(\text{C g}^{-1}) = \frac{i\Delta t}{m} \quad \text{(S1)}$$

$$\eta(\%) = \frac{\Delta t_D}{\Delta t_C} \times 100\% \quad \text{(S2)}$$

where i and m were discharging current (A) and mass of active substance (g), and Δt_C and Δt_D were the time (s) of charging and discharging at the same current.

To further evaluate the practical application potential, the hybrid supercapacitors (HSC, Co(Al)S//AC) were fabricated using optimal Co(Al)S as positive electrode and AC as negative electrode in a in a two-electrode system (3 mol L^{-1} KOH electrolyte), respectively. The AC electrode was prepared by mixing 80 wt% AC, 10 wt% carbon black and 10 wt% polyvinylidene fluoride onto the NF (1×1 cm^2) and then drying it. The GCD was tested on the CT2001A system (LANHE, Wuhan, China). The mass ratio of the two electrodes was determined by Eq. (S3), while power density (P, W kg^{-1}) and energy density (E, Wh kg^{-1}) of the HSC were calculated by Eqs. (S4) and (S5) [1,2],

$$\frac{m_+}{m_-} = \frac{C_{S-} \times \Delta V_-}{C_{S+} \times \Delta V_+} \quad \text{(S3)}$$

$$E = \frac{C_S \times V}{7.2} \quad \text{(S4)}$$

$$P = \frac{3600E}{\Delta t} \quad \text{(S5)}$$

Where C_s was the specific charge (C g^{-1}), ΔV was the potential window of electrode material (V), V was the operating potential of the device (V), Δt was the discharge time (s). m_+ and m_- were the mass of the positive and negative electrode active materials (g), respectively. The optimal mass ratio of the positive and negative electrodes was determined to be about 0.35 using the charge balance principle.

References

1. Zhang K., Zeng H.Y., Wang M. X., Li H.B., Yan W., Wang H.B., Tang Z.H. 3D hierarchical core-shell

structural NiCoMoS@NiCoAl hydrotalcite for high-performance supercapacitors. J. Mater. Chem. A, 2022, 10(20): 11213-11224.

2. Tang Z.H., Zeng H.Y., Zhang K., Yue H.L., Tang L.Q., Lv S.B., Wang H.B. Engineering core-shell NiC_2O_4@C/N-direct-doped NiCoZn-LDH for supercapacitors. Chem. Eng. Sci., 2024, 289: 119865.

Figure S1 Distribution of nanosheet thickness for the Co(Al)S based the SEM images.

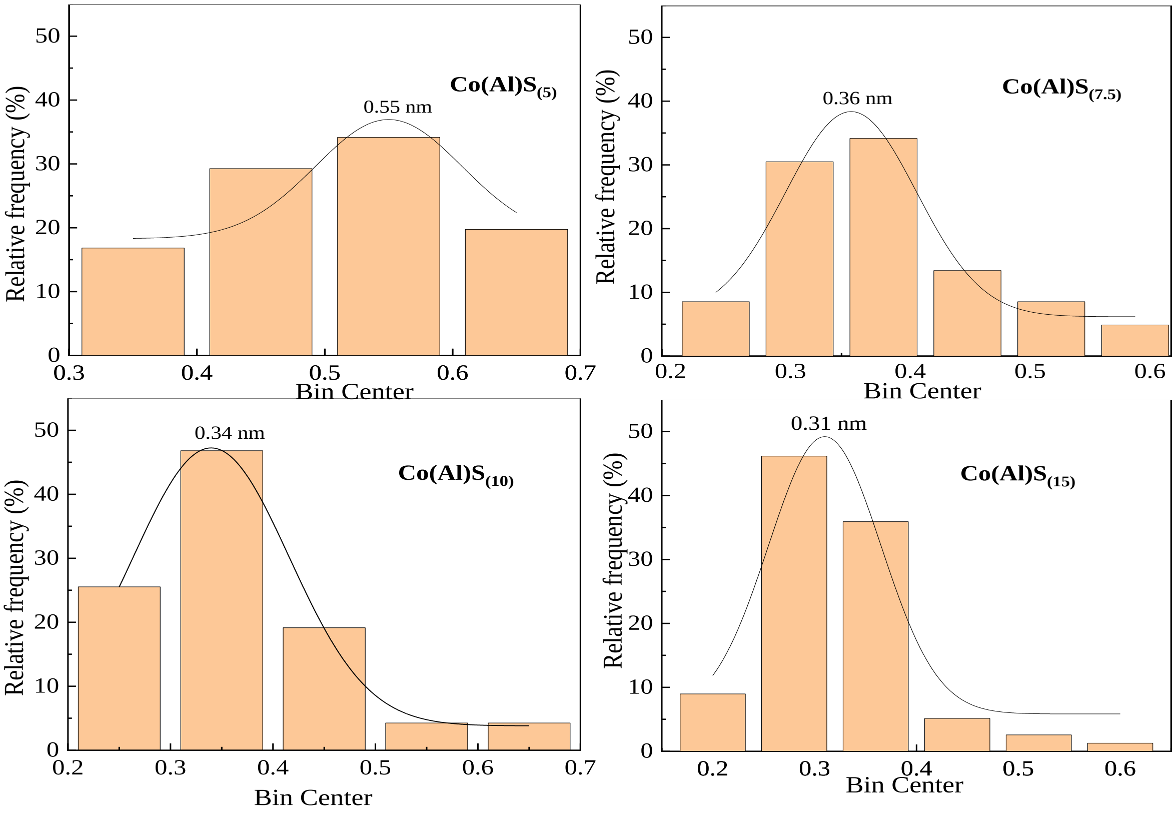

Figure S2 SEM-EDS pattern of the $Co(Al)S_{(10)}$ sample.

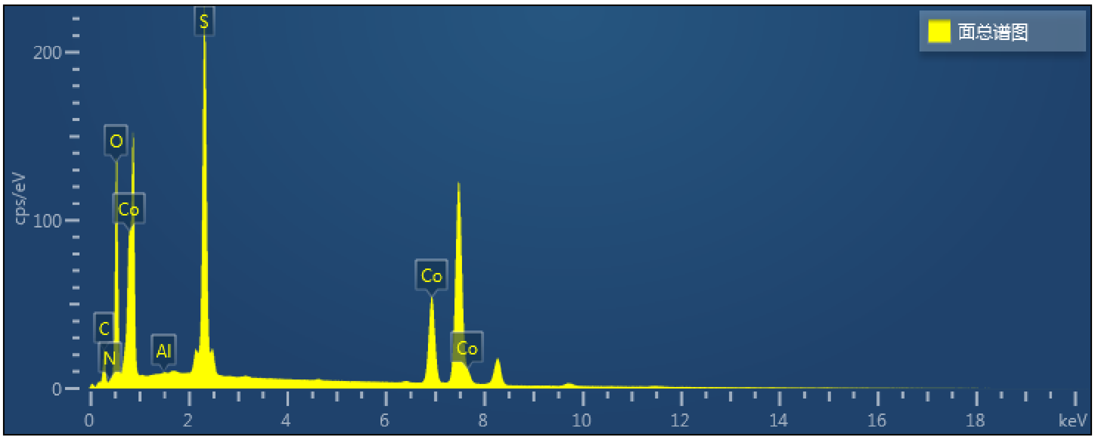

Figure S3 SEM and EDS-mapping images as well as the corresponding element distributions of the Co(Al)$S_{(10)}$ sample.

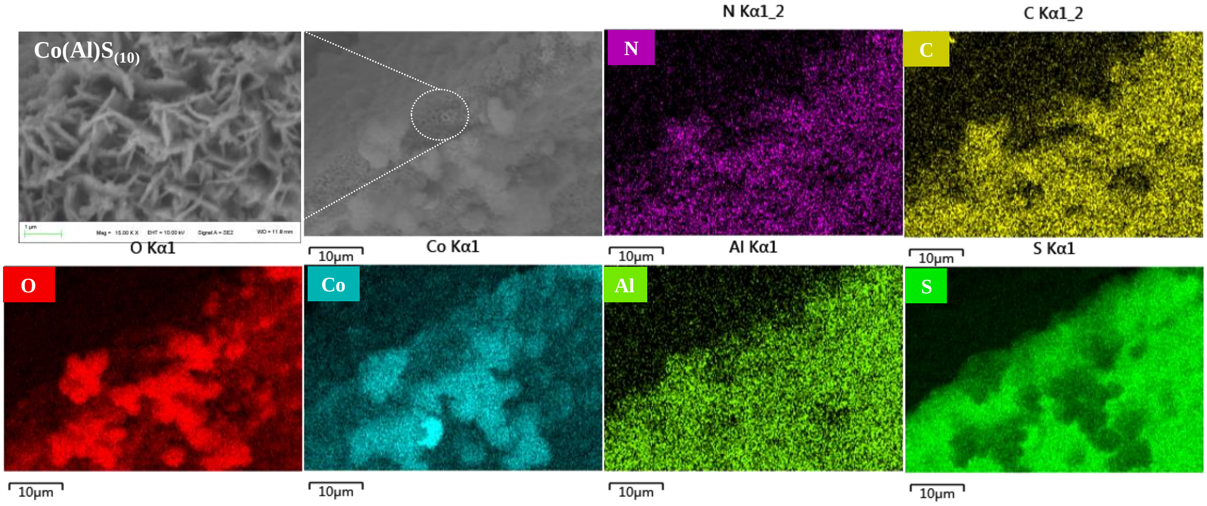

Figure S4 XRD patterns of the CAU-1 self-sacrificial template.

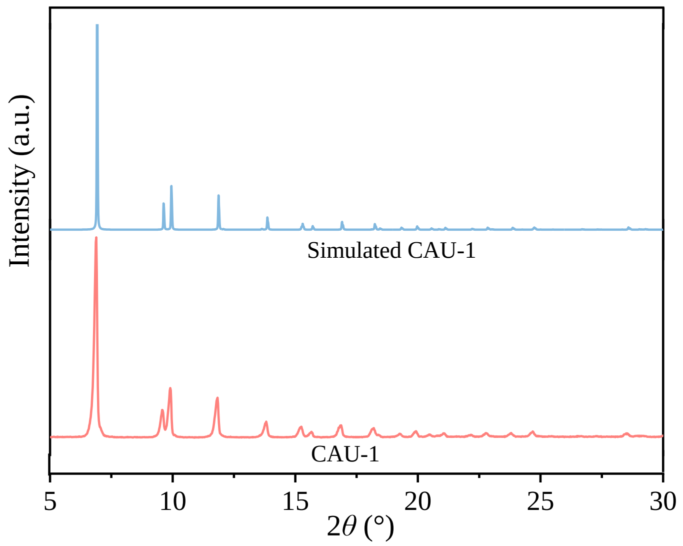

Figure S5 High-resolution O 1 s XPS spectrum of the Co(Al)S(10).

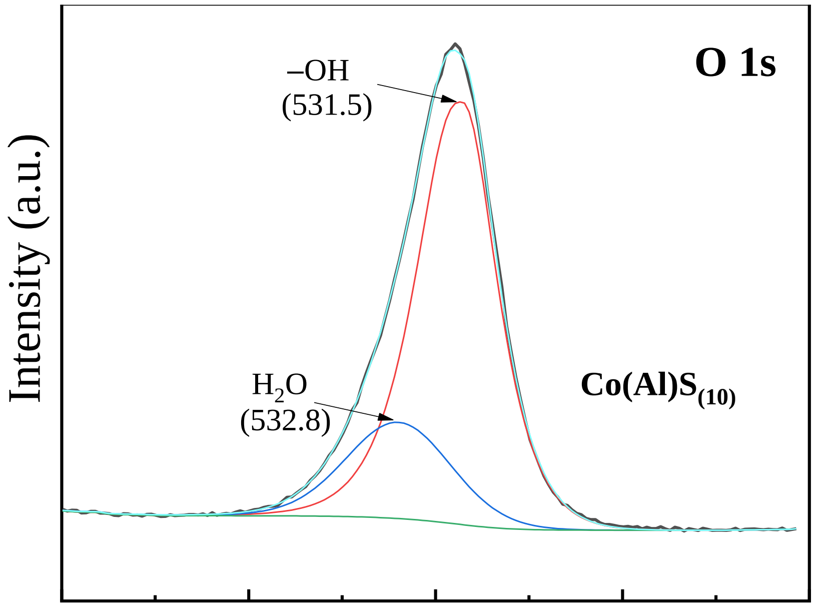

In the high-resolution O 1s spectra, the spectrum of the Co(Al)S(10) could be deconvoluted into three peaks at about 531.2, and 533.0 eV, corresponding to the surface −OH groups and surface adsorbed water (H_2O_{ads}), respectively [1-3]. The result confirmed that the O element in the Co(Al)S(10) was due to water molecules from the atmosphere, namely the Co(Al)S(10) had been fully transformed from $CoAl_2O_4$ intermediate into sulfides.

References

1. Zhang K., Zeng H. Y., Wang M. X., Li H. B., Yan W., Wang H. B., Tang Z. H. 3D hierarchical core-shell structural NiCoMoS@NiCoAl hydrotalcite for high-performance supercapacitors. J. Mater. Chem. A, 2022, 10(20): 11213-11224.

2. Ferrah D., Haines A.R., Galhenage R.P., Bruce J.P., Babore A.D., Hunt A., Waluyo I., Hemminger J.C. Wet chemical growth and thermocatalytic activity of Cu-based nanoparticles supported on TiO_2 nanoparticles/HOPG: in situ ambient pressure XPS study of the CO_2 hydrogenation reaction. ACS Catal., 2019, 9(8): 6783-6802.

3. Burghaus U. Surface chemistry of CO_2-Adsorption of carbon dioxide on clean surfaces at ultrahigh vacuum. Prog. Surf. Sci., 2014, 89(2): 161-217.

Figure S6 CV curves of the Co(Al)O, Co(Al)$S_{(5)}$, Co(Al)$S_{(7.5)}$ and Co(Al)$S_{(15)}$ at different scan rates.

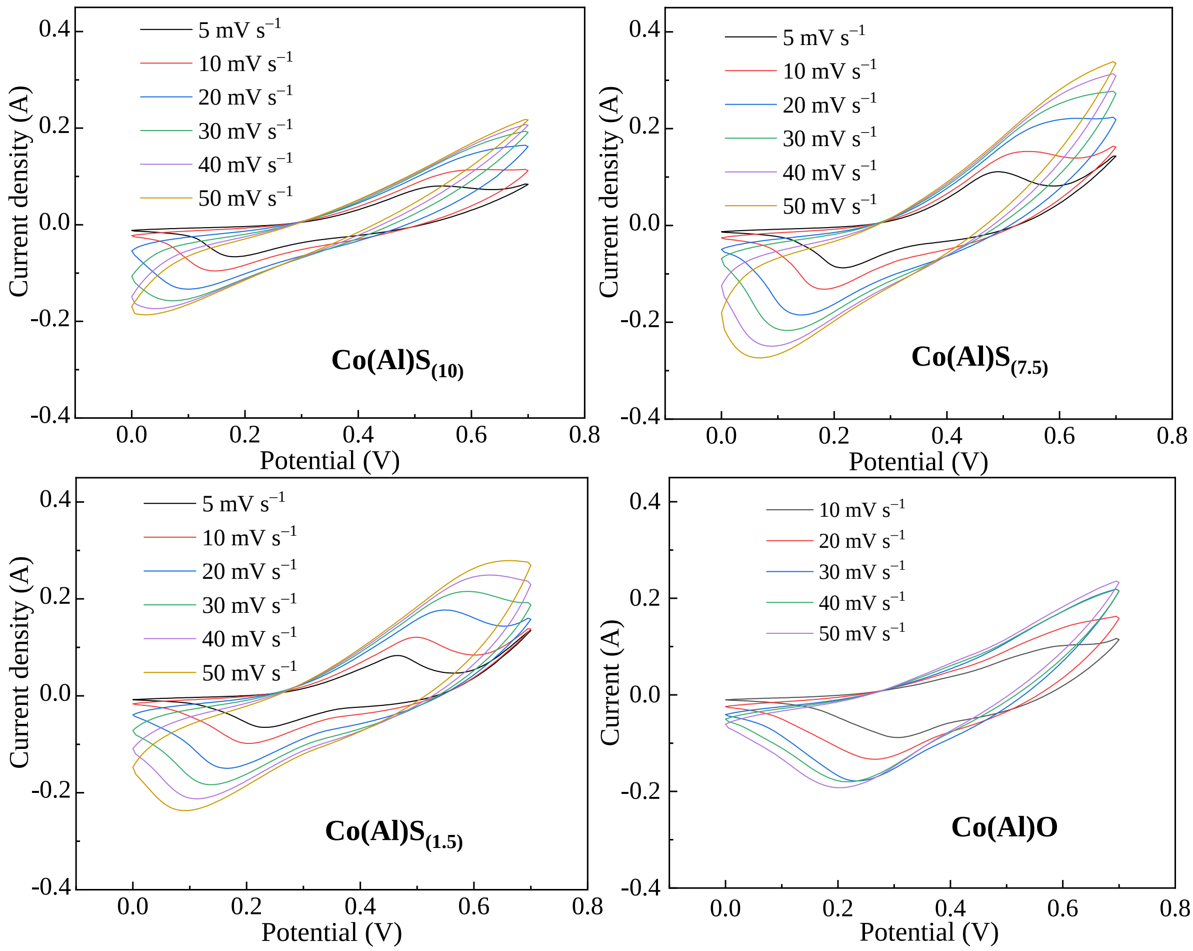

Figure S7 Randles-Sevcik plots of the peak current vs. square root of scan rate for the samples.

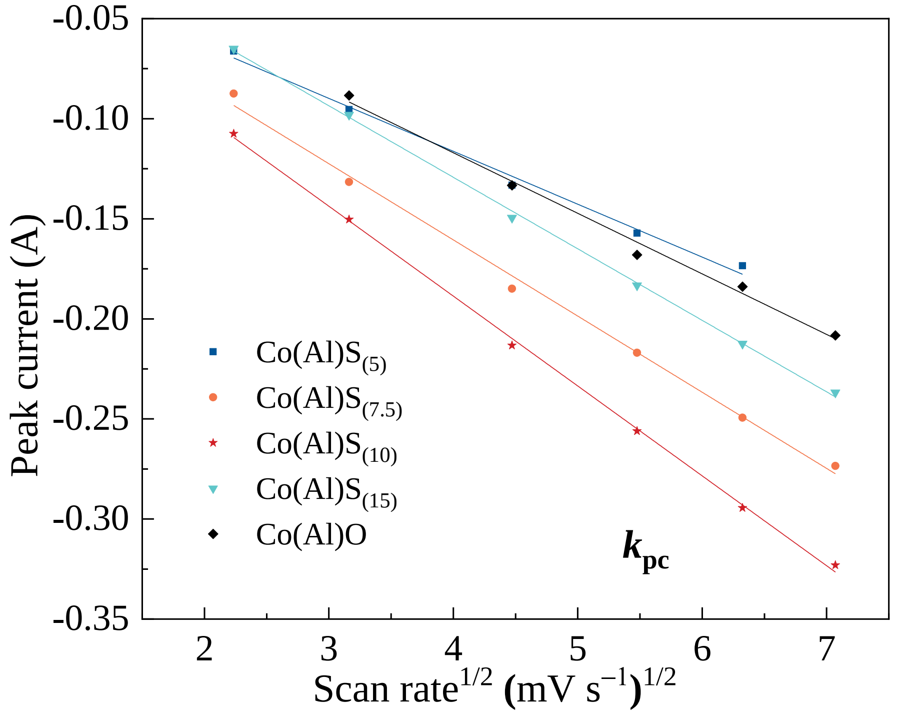

Figure S8 GCD profiles of the Co(Al)O, Co(Al)S(5), Co(Al)S(7.5) and Co(Al)S(15) at different current densities.

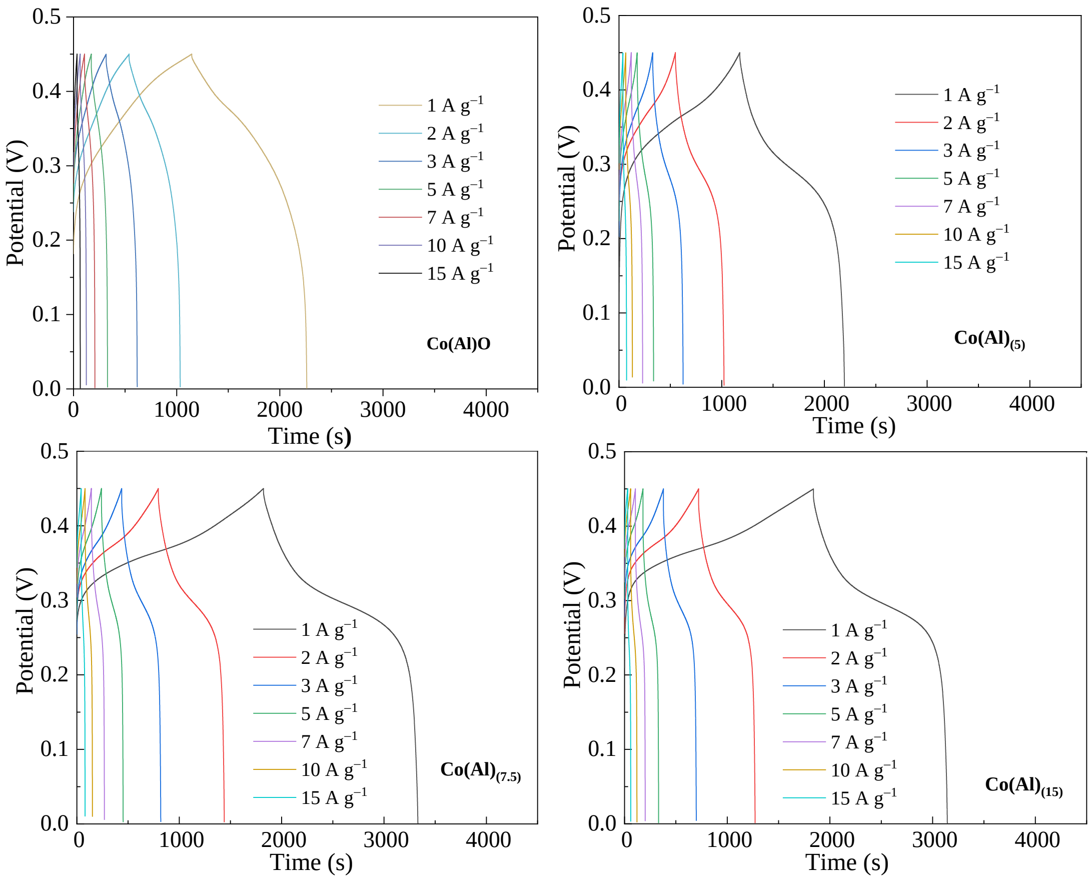

Table S1 Electrochemistry performances of the Co(Al)O and Co(Al)S.

	Area ($\times 10^{-2}$)	*b*	r^2_{pa}	i_{pc} (A)	CV kinetics		EIS		C_s ($C\ g^{-1}$, at $1\ A\ g^{-1}$)	Rate property (%, up to $15\ A\ g^{-1}$)
					k_{pc} (s^{-1})	R^2_{pc}	R_s (Ω)	R_{ct} (Ω)		
Co(Al)O	7.87	0.53	0.993	-0.178	-0.030	0.990	0.70	0.40	1118.0	55.2
Co(Al)$S_{(5)}$	5.41	0.47	0.994	-0.157	-0.026	0.996	0.69	0.41	1024.2	49.8
Co(Al)$S_{(7.5)}$	9.63	0.49	0.994	-0.217	-0.038	0.996	0.79	0.14	1506.1	37.6
Co(Al)$S_{(10)}$	12.21	0.52	0.998	-0.327	-0.046	0.949	0.66	0.13	1791.8	52.2
Co(Al)$S_{(15)}$	9.64	0.56	0.998	-0.183	-0.036	0.998	0.90	0.55	1305.6	41.1

* A_{area}: Integral area of CV curve; *b*: Adjustable parameter; i_{pc}: Peak current of cathode; k_{pc}: apparent reaction rates of cathode, respectively.

Table S2 Electrochemical properties of the Co(Al)S compared to the reported transition metal sulfides in the three-electrode system.

Electrode materials	KOH electrolyte (mol L^{-1})	Specific capacitance (C g^{-1})	Cycling stability	ref.
CuS_2@$CoNi_2S_4$	2	1191.6 (1 A g^{-1})	85.7% (10 000 cycles at 20 A g^{-1})	[1]
Ni-Ni_3S_4/CNTs*	1	744.9 (1 A g^{-1})	84.5% (3 000 cycles at 10 A g^{-1})	[2]
NiCoMn-S	1	1154.9 (1 A g^{-1})	-	[3]
$NiMoS_4$/NiS_2	3	1269.0 (1 A g^{-1})	80.0% (5 000 cycles at 10 A g^{-1})	[4]
NCM-S@HCNS*	2	1093.0 (1 A g^{-1})	90.4% (5 000 cycles at 10 A g^{-1})	[5]
NiCoS/CC*	6	900.6 (1 A g^{-1})	96.3% (1 000 cycles at 20 A g^{-1})	[6]
NiCoMoS@NiCoAl-LDH*	3	1336.0 (1 A g^{-1})	92.5% (2 000 cycles at 5 A g^{-1})	[7]
NiCoMnS/NF	3	872.0 (1 A g^{-1})	87.7% (2 000 cycles at 5 A g^{-1})	[8]
FC@MS-EG*	3	1956.0 (1 A g^{-1})	91.1% (6 000 cycles at 8 A g^{-1})	[9]
FM-Co_3S_4*	6	1404.0 (5 A g^{-1})	88.9% (5 000 cycles at 10 A g^{-1})	[10]
Co(Al)$S_{(10)}$	3	1791.8 (1 A g^{-1})	88.4% (5 000 cycles at 1 A g^{-1})	This work

*: CNTs: Carbon nanotubes; NCM-S: Ni-Co-S/crystalline MnS; HCNS: hollow carbon nanospheres; CC: Carbon cloth; LDH: layered double hydroxides; FC: FeCo-based nanoneedles; MS-EG: amorphous MoS_x nanoparticles; FM-Co_3S_4: Fe and Mn co-doped Co_3S_4.

Reference

1. Hao C., Ni C., Wang X., Pan Y., Wu Q., Wu J., Wang X. Fabrication of three-dimensional CuS_2@$CoNi_2S_4$ core-shell rod-like structures as cathode and thistle-derived carbon as anode for hybrid supercapacitors. Chem. Eng. J., 2023, 465: 143024.
2. Yang Y., Li M. L., Lin J.N., Zou M.Y., Gu S.T., Hong X.J., Si L.P., Cai Y.P. MOF-derived Ni_3S_4 encapsulated in 3D conductive

network for high-performance supercapacitor. Inorg. Chem., 2020, 59(4): 2406-2412.

3. Kang C., Ma L., Chen Y., Fu L., Hu Q., Zhou C., Liu Q. Metal-organic framework derived hollow rod-like NiCoMn ternary metal sulfide for high-performance asymmetric supercapacitors. Chem. Eng. J., 2022, 427: 131003.
4. Huang B., Yuan J., Lu Y., Zhao Y., Qian X., Xu H., He G., Chen H. Hollow nanospheres comprising amorphous $NiMoS_4$ and crystalline NiS_2 for all-solid-state supercapacitors. Chem. Eng. J., 2022, 436: 135231.
5. Zhou Y., Jia Z., Zhao S., Chen P., Wang Y., Guo T., Wei L., Cui X., Ouyang X., Wang X., Zhu J., Sun J., Pan S., Fu, Y. Construction of triple-shelled hollow nanostructure by confining amorphous Ni-Co-S/crystalline MnS on/in hollow carbon nanospheres for all-solid-state hybrid supercapacitors. Chem. Eng. J., 2021, 416: 129500.
6. Tang X., Wang J., Zhang D., Wang B., Xia X., Meng X., Yang B., Chen J., He Y., Han Z. In-situ construction of carbon cloth-supported amorphous/crystalline hybrid NiCo-sulfide with permeable concrete-like morphology for high-performance solid-state hybrid supercapacitors. Chem. Eng. J., 2023, 452: 139429.
7. Zhang K., Zeng H. Y., Wang M. X., Li H. B., Yan W., Wang H. B., Tang Z. H. 3D hierarchical core-shell structural NiCoMoS@NiCoAl hydrotalcite for high-performance supercapacitors. J. Mater. Chem. A, 2022, 10(20): 11213-11224.
8. Yan W., Zeng H.Y., Zhang K., Long Y.W., Wang M.X. Ni-Co-Mn hydrotalcite-derived hierarchically porous sulfide for hybrid supercapacitors. J. Colloid Interf. Sci., 2023, 635: 379-390.
9. Zhao Y., Wang S., Yuan M., Chen Y., Huang Y., Lian J., Yang S., Li H., Wu L. Amorphous MoS_x nanoparticles grown on cobalt-iron-based needle-like array for high-performance flexible asymmetric supercapacitor. Chem. Eng. J., 2021, 417: 127927.
10. Lu W., Yang Y., Zhang T., Ma L., Luo X., Huang C., Ning J., Zhong Y., Hu Y. Synergistic effects of Fe and Mn dual-doping in Co_3S_4 ultrathin nanosheets for high-performance hybrid supercapacitors. J. Colloid Interf. Sci., 2021, 590: 226-237.
